# Supplementary material for: Manipulating directional flow in a two-dimensional photonic quantum walk under a synthetic magnetic field
Source: Nat Commun. 2023 Oct 7;14:6283. doi: 10.1038/s41467-023-42045-4 (PMC10560269; doi:10.1038/s41467-023-42045-4)
Supplement: Supplementary file 1 — Supplemental Information [file 41467_2023_42045_MOESM1_ESM.pdf]

SUPPLEMENTAL INFORMATION FOR "MANIPULATING DIRECTIONAL FLOW IN A TWO-DIMENSIONAL PHOTONIC QUANTUM WALK UNDER A SYNTHETIC MAGNETIC FIELD"

In this Supplemental information, we provide numerical results characterizing the non-Hermitian skin effect (NHSE) and the Floquet topological invariants. We also clarify the impact of magnetic confinement and dynamics for $\alpha \in [0.5, 1]$.

Supplementary Note 1 - Quasienergy spectra

The presence of NHSE can be confirmed by examining the quasienergy spectra of the effective Hamiltonian under different boundary conditions, and the spatial distribution of eigenstates under the open boundary condition. As discussed in the main text (Methods section), we define the effective Hamiltonian $H_{\text{eff}} = i \ln U$, where U is the Floquet operator in Eq. (1) of the main text, and the branch cut of the logarithm is taken to be the negative real axis. The quasienergy spectrum of H_{eff} thus lies within the range $[-\pi, \pi)$.

In Supplementary Figures 1**a** and **c**, we show the quasienergy spectra under both the periodic (grey) and open (red) boundary conditions. The collapse of the spectra, when the boundary condition is changed from periodic to open, strongly suggests the presence of the NHSE. This is explicitly confirmed in Supplementary Figures 1**b** and **d**, where we show the spatial distribution of the eigenstates under the open boundary condition. They accumulate to

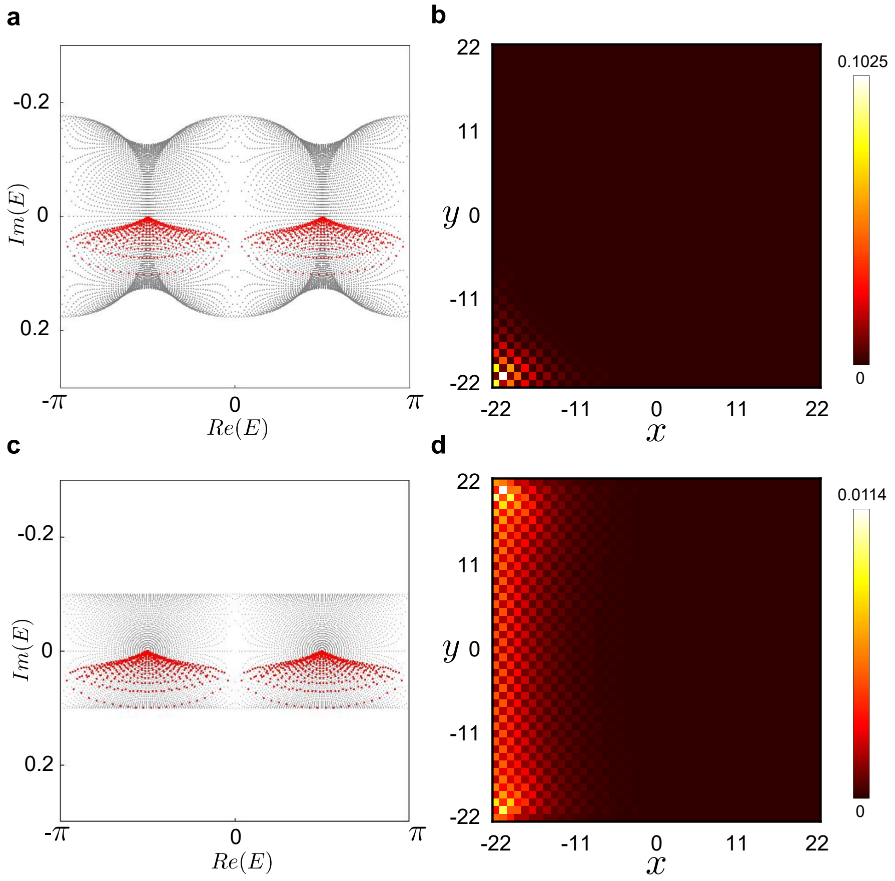

Supplementary Figure 1. **a** Numerically calculated quasienergy spectra of H_{eff} on the complex plane, with $\gamma_x = \gamma_y = 0.125$, under either the periodic boundary condition (grey) or the open boundary condition (red). **b** Spatial distribution of the eigenstates under the open boundary condition, with the same parameters used in **a**. **c** Numerically calculated quasienergy spectra of H_{eff} with $\gamma_x = 0.1$, $\gamma_y = 0$, under the periodic boundary condition (grey) and the open boundary condition (red), respectively. **d** Spatial distribution of the eigenstates under the open boundary condition, with the same parameters used in **c**. For numerical calculations, a lattice size of 45×45 is taken as an example, with $\alpha = 0$.

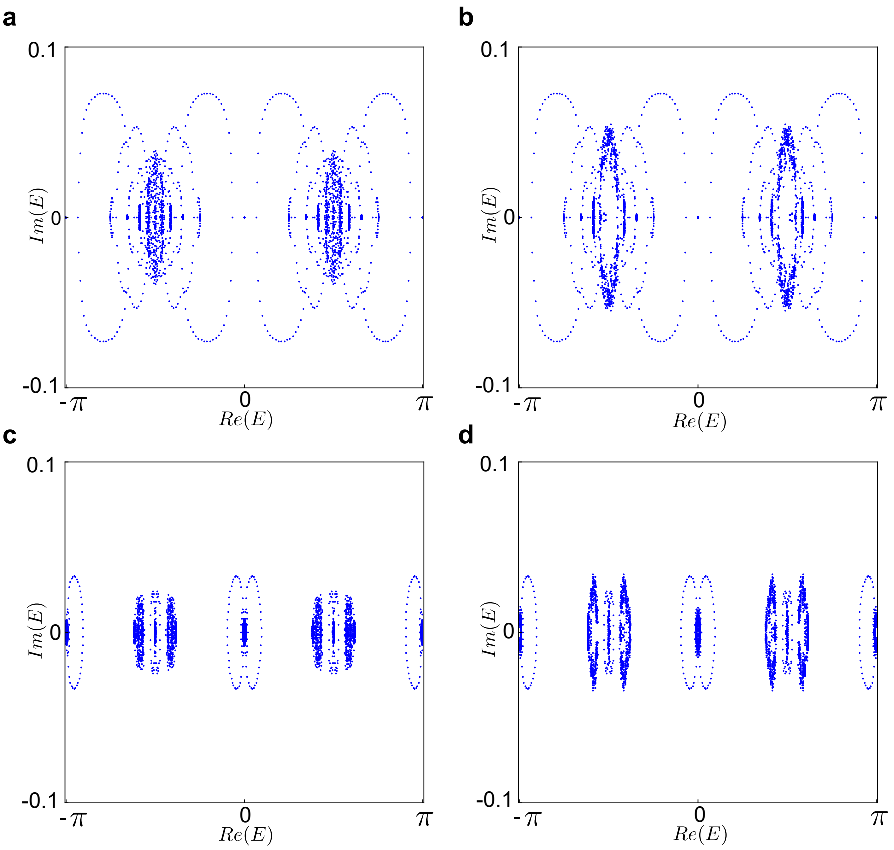

Supplementary Figure 2. Numerically calculated quasienergy spectra of H_{eff} on the complex plane under the periodic boundary condition. **a-d** The parameters are the same as those of Fig. 3**a-d** in the main text, respectively.

one edge or one corner, depending on the loss parameters. Note that H_{eff} and U share the same set of eigenstates. In Supplementary Figure 2 and Supplementary Figure 3, we show the quasienergy spectra corresponding to Fig. 3 and Fig. 4 of the main text, respectively.

As a function of the synthetic flux, the quasienergy spectrum exhibits an intricate fractal-like pattern as shown in Supplementary Figure 4. There are altogether $2q$ bands, indicating the spin multiplicity and the size of the magnetic unit cell for each rational value of the flux $\alpha = p/q$. We find that the quasienergy spectrum exhibits typical forms of the Floquet Hofstadter butterfly under all our experimental parameters. Under the influence of NHSE, the energy gaps will close when γ increases to a sufficiently large value (Supplementary Figure 4**c**).

Supplementary Note 2 - Impact of magnetic confinement

The competition between the non-Hermitian skin effect and the magnetic confinement refers to the boundary localization under the non-Hermitian skin effect, versus the magnetic confinement that leads to localized eigenstates in the bulk. In terms of bulk dynamics, this gives rise to the suppressed directional flow as observed in our experiment.

As illustrated in Fig. 1**a** of the main text, the walker acquires a position-dependent phase when moving along the y direction, thanks to the operator sequence S_yP in the Floquet operator U. This process contributes a position-dependent phase to the hopping terms in the high-frequency effective Hamiltonian (see discussions on the approximate tight-binding model in Methods). As a consequence, when the walker goes around the plaquette in a unit cell, it accumulates a non-vanishing phase $2\pi\alpha$. The position-dependent phase can be identified as the Peierls phase, which appears in the hopping terms of charged particles under a magnetic field [1, 2]. As such, our quantum-walk dynamics is akin to the dynamics of a charged particle moving in a two-dimensional lattice, perpendicular to a uniform magnetic field. The synthetic flux α then corresponds to the magnetic flux threaded through each unit cell.

Now, for a charged particle moving in a plane perpendicular to a uniform magnetic field, it experiences a force

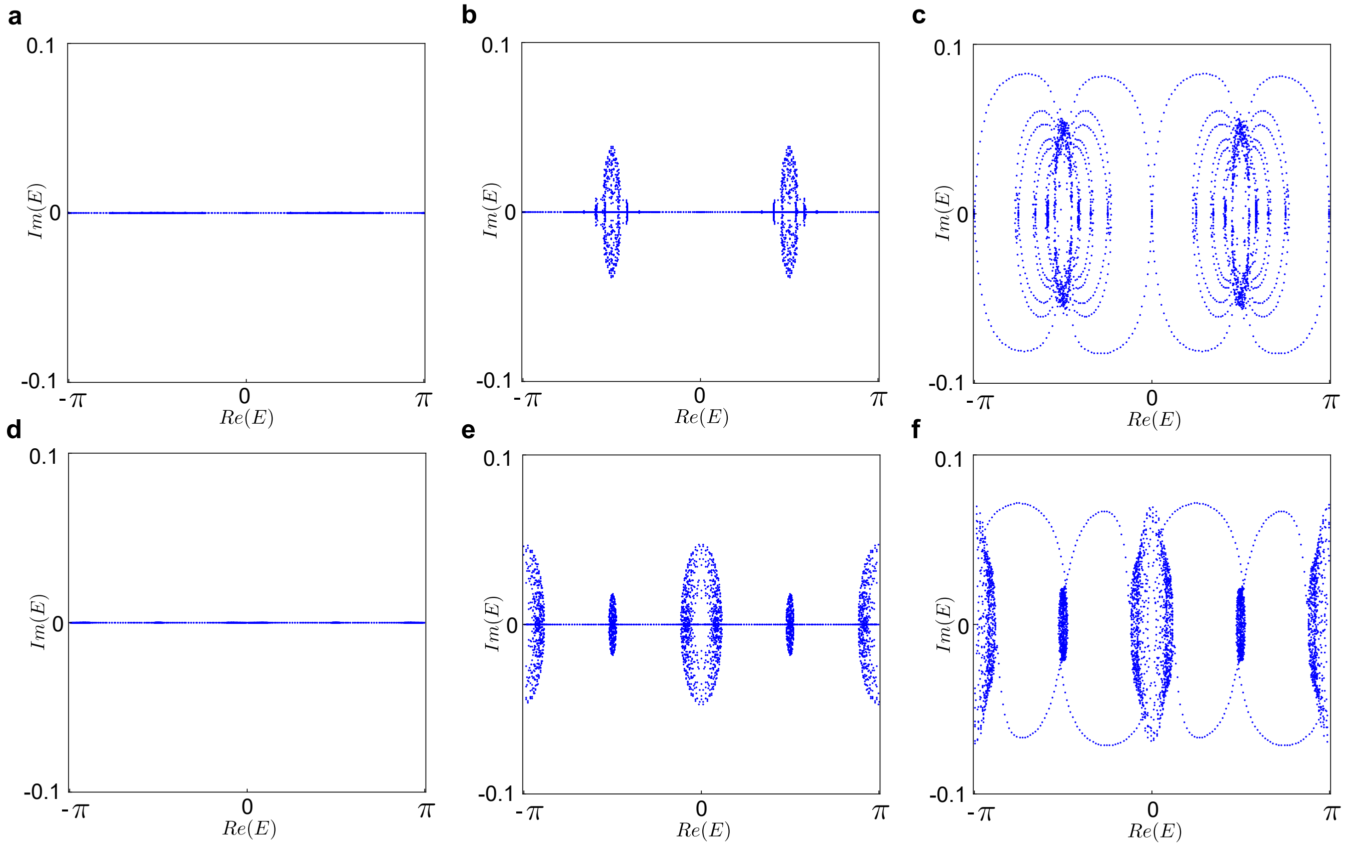

Supplementary Figure 3. Numerically calculated quasienergy spectra of $H_{\rm eff}$ on the complex plane under the domain-wall geometry. **a-f** The parameters are the same as those of Fig. 4**a-f** in the main text, respectively.

perpendicular to both its direction of motion and the magnetic field, leading to cyclotron motion with localized orbits. The quantization of these cyclotron orbits gives flat Landau bands with localized eigenstates [3, 4]. Since such a localization occurs in the bulk, it is incompatible, and and competes with the non-Hermitian skin effects which deform the eigenstates toward the boundary. The dynamic manifestation of such a competition is the flux-induced suppression of the direction flow, as we reported in the experiment.

To gain further insight into the competition, we plot the quasienergy spectra under different magnetic fluxes in Supplementary Figure 5. For this purpose, we define the effective Hamiltonian through $U = e^{-iH_{\rm eff}}$, and calculate the eigenspectra of $H_{\rm eff}$. When $\alpha = 0$, the eigenspectra occupy a finite area in the complex plane under the periodic boundary condition, whereas the eigenspectra collapse (occupying zero area) under the open boundary condition. The spectral topology under the periodic boundary condition is a key indicator of the non-Hermitian skin effect in two dimensions. Dynamically, the area occupied by the eigenspectra corresponds to the short-time acceleration of the directional flow [5]. However, under a finite α, such an area shrinks significantly (see Supplementary Figure 5**b**), indicating the flux-induced suppression of the directional flow, as well as the non-Hermitian skin effect.

The observation is further corroborated by the spatial distribution of eigenstates. We define the averaged spatial distribution of eigenstates under the open boundary condition

$$\overline{|\psi(x)|} = \sum_{i}^{Ne} \sum_{y} \frac{|\psi_i(x,y)|^2}{Ne}, \tag{1}$$

$$\overline{|\psi(y)|} = \sum_{i}^{Ne} \sum_{x} \frac{|\psi_i(x,y)|^2}{Ne}, \tag{2}$$

where $|\psi_i(x,y)|^2$ is the occupation of the ith eigenstate at site (x,y), and Ne is the total number of eigenstates. As shown Supplementary Figure 5(c)(d), while the non-Hermitian skin effect is apparent in either spatial direction, it is suppressed by the presence of a magnetic flux. Our observed competition between the non-Hermitian skin effect and

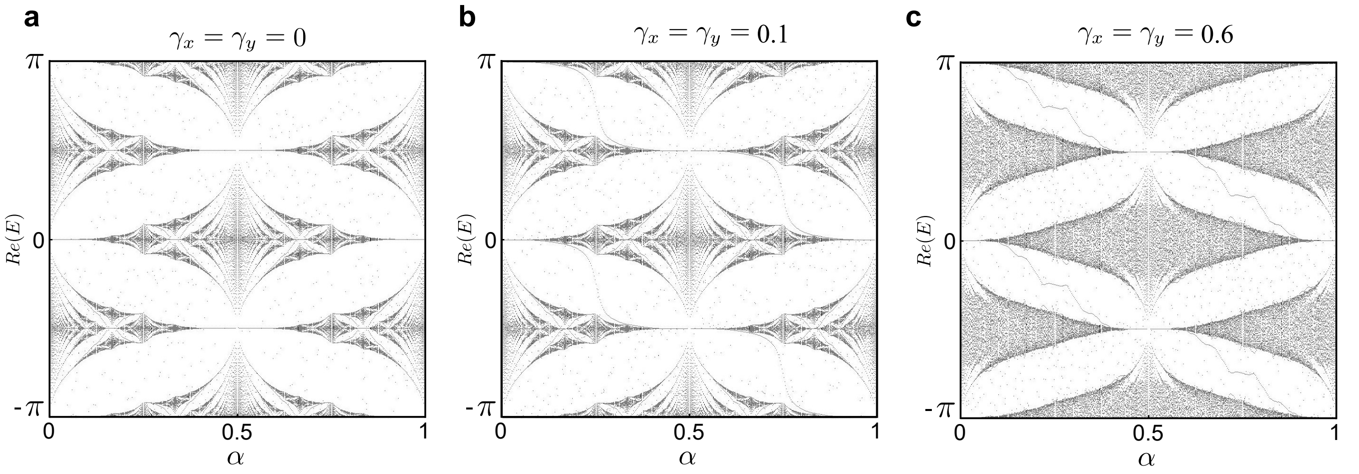

Supplementary Figure 4. The real components of energy spectrum plotted against rational values of the synthetic flux α under different γ. The loss parameters are chosen as $\gamma_x = \gamma_y = 0$ in **a**, $\gamma_x = \gamma_y = 0.1$ in **b** and $\gamma_x = \gamma_y = 0.6$ in **c**, respectively. For numerical calculations, we take a lattice size $N = 89$ in the x dimension.

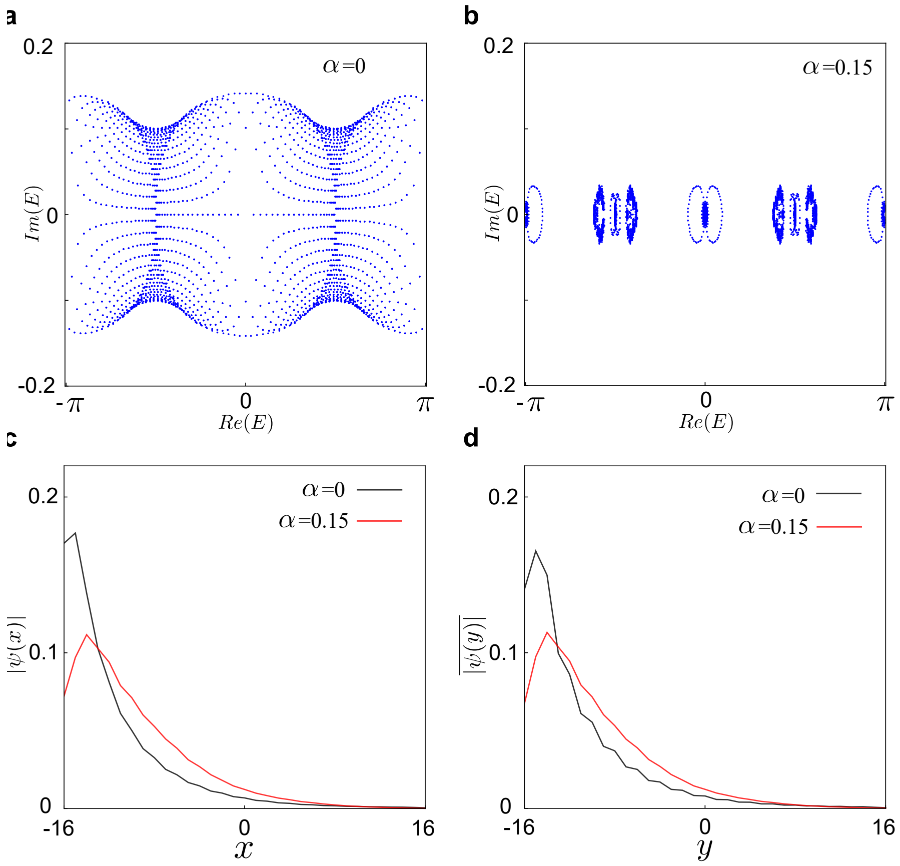

Supplementary Figure 5. **a,b** Numerically calculated quasienergy spectra of H_{eff} on the complex plane under the periodic boundary condition with $\gamma_x = \gamma_y = 0.1$ under different α. **c,d** The corresponding averaged spatial distribution of eigenstate in x and y directions under the open boundary condition. For numerical calculations, we take a lattice with 33×33 sites.

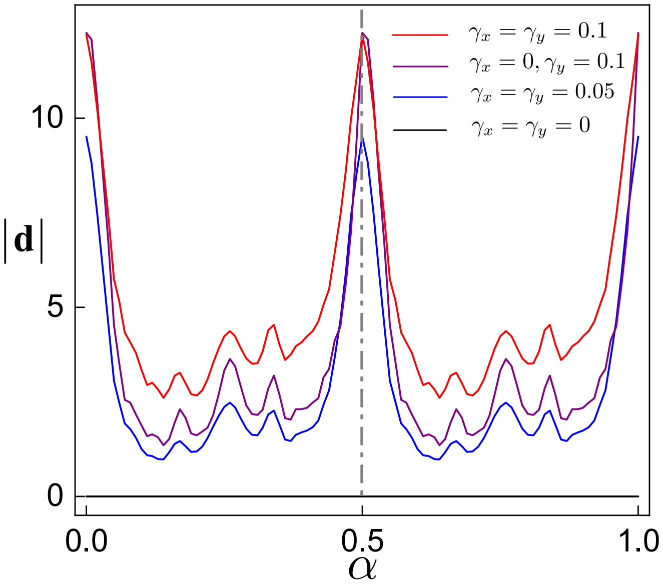

Supplementary Figure 6. Numerically evaluated norm of the directional displacement $\mathbf{d}$ for 16-time-step quantum walks, with $\alpha \in [0, 1]$. The parameters are the same as those in Fig. 3(e) of the main text. The gray line marks $\alpha = 0.5$.

the magnetic confinement is also consistent with previous theoretical studies [4, 6].

Supplementary Note 3 - Dynamics of α in the range $[0.5, 1]$

In the experiment, we choose the parameter range $\alpha \in [0, 0.5)$ because the dynamics of on-site occupation under $U(\alpha)$ and $U(\alpha + 0.5)$ are the same. To explicitly demonstrate this, we extend the parameter range of Fig. 3**e** in the main text, and show the numerically evaluated $|\mathbf{d}|$ in Supplementary Figure 6, with the observation that $|\mathbf{d}(\alpha)| = |\mathbf{d}(\alpha + 0.5)|$.

To understand this observation, we fit the Floquet operator U in a different time frame, that is, subject it to a unitary transformation $\overline{U} = S_y^\dagger U S_y$, where S_y is the shift operator defined in the main text. Observables such the directional displacement are not changed under different time frames. Denoting $U_0 = M_y C M_x S_x C$, we have

$$\begin{aligned}\overline{U} &= P U_0 \\ &= P_{\text{odd}} U_0 + P_{\text{even}} U_0,\end{aligned} \tag{3}$$

where

$$P_{\text{odd (even)}} = \begin{cases} \sum_{x,y} \begin{pmatrix} e^{i2\pi\alpha x} & 0 \\ 0 & e^{-i2\pi\alpha x} \end{pmatrix} \otimes |x, y\rangle \langle x, y|\,, & x \text{ is odd (even);} \\ 0, \quad \text{otherwise.} \end{cases} \tag{4}$$

Consider $\alpha = \alpha + 0.5$, we have

$$\overline{U}(\alpha) = P_{\text{odd}}(\alpha) U_0 + P_{\text{even}}(\alpha) U_0, \tag{5}$$

$$\overline{U}(\alpha') = -P_{\text{odd}}(\alpha) U_0 + P_{\text{even}}(\alpha) U_0. \tag{6}$$

Under the specific design of the shift operator S_x, U_0 would transform state components on lattice sites with odd-x label to a superposition of its neighboring sites with even-x label, and vice versa. Hence, given the same initial states, the time-evolved states under $\overline{U}(\alpha)$ and $\overline{U}(\alpha')$ would differ only by local signs, which do not change the probability distribution over the lattice sites. For instance, in the experimentally relevant case of even number of time steps t, the time-evolved states under $\overline{U}(\alpha)$ and $\overline{U}(\alpha')$ would only differ by a global phase $(-1)^{\frac{t}{2}}$, which does not change the occupation distribution.

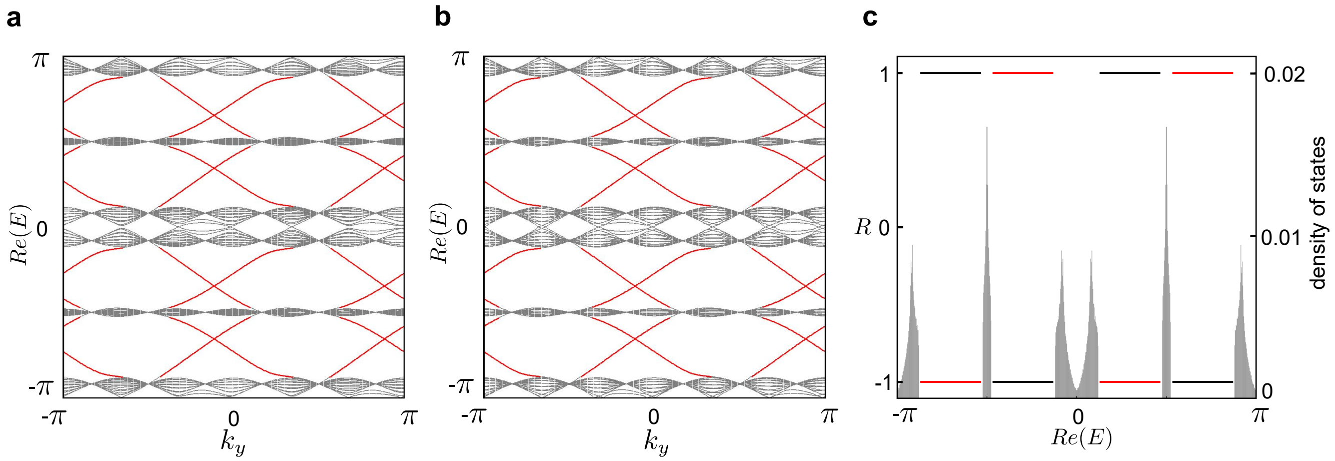

Supplementary Figure 7. **a, b** The real components of the quasienergy spectra under the domain-wall geometry of Figs. 4**d** and **f** in the main text, with $\gamma_x = \gamma_y = 0$ in **a**, and $\gamma_x = \gamma_y = 0.1$ in **b**. Floquet topological edge states (red) are seen to emerge within each quasienergy gap. **c** Floquet topological invariants R as functions of the real component of the quasienergy. Within each quasienergy gap, the red (black) line indicates R of the left (right) region. The shaded areas are the density of states normalized to the total number of states, indicating the regions of quasienergy bands. For numerical calculations, we take a lattice size with 45×45, and fix $\alpha = 1/3$ and $\alpha = -1/3$ for the left and right regions, respectively.

Supplementary Note 4 - Floquet topological invariants and edge states

In Supplementary Figures 7**a** and **b**, we show the real components of the quasienergy spectra under the domain-wall geometry, respectively under the parameters of Fig. 4**d** and Fig. 4**f** in the main text. While the Floquet topological edge states are visible within each quasienergy gap, the quasienergy spectra are close to each other, because of the smallness of the loss parameters in Supplementary Figure 7**b**.

The Floquet topological edge states can be characterized by the gap invariant R defined in the Methods section of the main text. In Supplementary Figure 7**c**, we show the calculated gap invariants R for the left (red) and right (black) regions of the domain-wall configuration in Supplementary Figure 7**b**. The topological invariants of the two regions are always finite but differ by their signs. Importantly, within each quasienergy gap, the difference in the gap topological invariants between the two regions is 2. This corresponds to the number of Floquet topological edge states within each quasienergy gap, as illustrated in Supplementary Figure 7**b**.

[1] Peierls, R. Zur theorie des diamagnetismus von leitungselektronen. *Z. Phys.* **80**, 763 (1933).
[2] Yalnkaya, İ. & Gedik, Z. Two-dimensional quantum walk under artificial magnetic field. *Phys. Rev. A* **92**, 042324 (2015).
[3] Aoki, H., Ando, M. & Matsumura, H. Hofstadter butterflies for flat bands. *Phys. Rev. B* **54**, R17296 (1996).
[4] Shao, K., Cai, Z.-T., Geng, H., Chen, W. & Xing, D. Cyclotron quantization and mirror-time transition on nonreciprocal lattices. *Phys. Rev. B* **106**, L081402 (2022).
[5] Longhi, S. Non-Hermitian skin effect and self-acceleration. *Phys. Rev. B* **105**, 245143 (2022).
[6] Lu, M., Zhang, X.-X. & Franz, M. Magnetic suppression of non-Hermitian skin effects. *Phys. Rev. Lett.* **127**, 256402 (2021).
